# The Impact of Different Degrees of Intraventricular Hemorrhage on Mortality and Neurological Outcomes in Very Preterm Infants: A Prospective Cohort Study

**DOI:** 10.3389/fneur.2022.853417

**Published:** 2022-03-21

**Authors:** Yong Wang, Juan Song, Xiaoli Zhang, Wenqing Kang, Wenhua Li, Yuyang Yue, Shan Zhang, Falin Xu, Xiaoyang Wang, Changlian Zhu

**Affiliations:** ^1^Henan Key Laboratory of Child Brain Injury and Henan Pediatric Clinical Research Center, Institute of Neuroscience and Third Affiliated Hospital of Zhengzhou University, Zhengzhou, China; ^2^Department of Neonatology, Children's Hospital of Zhengzhou University, Zhengzhou, China; ^3^Center for Perinatal Medicine and Health, Institute of Neuroscience and Physiology, Sahlgrenska Academy, University of Gothenburg, Gothenburg, Sweden; ^4^Center for Brain Repair and Rehabilitation, Institute of Neuroscience and Physiology, University of Gothenburg, Sahlgrenska Academy, Gothenburg, Sweden

**Keywords:** very preterm infants, intraventricular hemorrhage, mortality, neurological disability, outcomes

## Abstract

**Objective:**

Intraventricular hemorrhage (IVH) is a common complication in preterm infants and is related to neurodevelopmental outcomes. Infants with severe IVH are at higher risk of adverse neurological outcomes and death, but the effect of low-grade IVH remains controversial. The purpose of this study was to evaluate the impact of different degrees of IVH on mortality and neurodevelopmental outcomes in very preterm infants.

**Methods:**

Preterm infants with a gestational age of <30 weeks admitted to neonatal intensive care units were included. Cerebral ultrasound was examined repeatedly until discharge or death. All infants were followed up to 18–24 months of corrected age. The impact of different grades of IVH on death and neurodevelopmental disability was assessed by multiple logistic regression.

**Results:**

A total of 1,079 preterm infants were included, and 380 (35.2%) infants had grade I-II IVH, 74 (6.9%) infants had grade III-IV IVH, and 625 (57.9%) infants did not have IVH. The mortality in the non-IVH, I-II IVH, and III-IV IVH groups was 20.1, 19.7, and 55.2%, respectively (*p* < 0.05), and the incidence of neurodevelopmental disabilities was 13.9, 16.1, and 43.3%, respectively (*p* < 0.05), at 18–24 months of corrected age. After adjusting for confounding factors, preterm infants with III-IV IVH had higher rates of cerebral palsy [26.7 vs. 2.4%, OR = 6.10, 95% CI (1.840–20.231), *p* = 0.003], disability [43.3 vs. 13.9%, OR = 2.49, 95% CI (1.059–5.873), *p* = 0.037], death [55.2 vs. 20.1%, OR = 3.84, 95% CI (2.090–7.067), *p* < 0.001], and disability + death [73.7 vs. 28.7%, OR = 4.77, 95% CI (2.518–9.021), *p* < 0.001] compared to those without IVH. However, the mortality and the incidence of neurodevelopmental disability in infants with I-II IVH were similar to those without IVH (*p* > 0.05).

**Conclusions:**

Severe IVH but not mild IVH increased the risk of mortality and neurodevelopmental disability in very preterm infants.

## Introduction

Despite advances in the care of newborns, intraventricular hemorrhage (IVH) remains a common complication in preterm infants, especially in those <32 weeks' gestation (1). The overall incidence of IVH is about 36% in preterm infants (2), and the incidence of IVH increases as gestational age and birth weight decrease (3). It occurs in about 45% of extremely preterm infants with a birth weight of <750 g and in about 52% of preterm infants born at <28 weeks' gestation (4, 5). About 50% of cases of IVH occur within 1 day after birth, and about 90% occur within 3 days of life (6).

IVH is graded from I to IV according to the classification of Papile (7). Several studies have shown that III-IV IVH implies more severe pathologic injuries and that it is associated with greater adverse neurological outcomes in preterm infants (8–11) such as cerebral palsy (CP), intellectual ability, and impairment of academic skills. Although I-II IVH is generally considered benign, subtle impairment in subcortical white matter and decreasing cortical gray matter following I-II IVH have been identified by cerebral MRI and might influence the development of processing skills and motor coordination (12, 13). Whether I-II IVH leads to poor neurodevelopmental outcomes in preterm infants remains a controversial issue (14–17), and the purpose of this study was to assess the impact of different grades of IVH (grade I-II IVH and grade III-IV IVH) on neurodevelopmental outcomes and death in very preterm infants.

## Methods

### Study Population

This was a prospective cohort study of preterm infants with a gestational age <30 weeks who were admitted to the NICU in the Third Affiliated Hospital and Children's Hospital of Zhengzhou University between July 2012 and December 2019. All infants were screened by cranial ultrasound within 3 days after birth, then on day 7, and then weekly until discharge. The diagnosis and classification of IVH were based on those of Papile (7). Grade I was defined as germinal matrix hemorrhage, grade II was defined as intraventricular hemorrhage without ventricular dilatation, grade III was defined as intraventricular hemorrhage filling more than 50% of the ventricle and with ventricular dilatation (which refers to ventricular index values > 97th percentile), and grade IV was defined as intraparenchymal hemorrhage. Preterm infants who had congenital or genetic diseases, congenital cranial malformation, who died before cranial ultrasound examination, or who refused to participate were excluded. This study was approved by the Ethics Committee of the Third Affiliated Hospital of Zhengzhou University, and informed consent was signed by all parents.

### Clinical Characteristics

The following factors were considered as potentially impacting on neurological disabilities and death in preterm infants: neonatal characteristics (gestational age, birth weight, gender, 5 min Apgar score, small for gestational age, delivery mode, and twin/multiple births), maternal characteristics (pregnancy hypertension, maternal age ≥35 years, placental abruption, gestational diabetes, abnormal amniotic fluid, fetal distress, and premature rupture of membranes), medical treatments (mechanical ventilation >7 days and erythropoietin treatment), and neonatal complications (respiratory distress syndrome (RDS), necrotizing enterocolitis (NEC), bronchopulmonary dysplasia (BPD), periventricular leukomalacia (PVL), sepsis, and severe anemia).

Small for gestational age is defined as birth weight below the 10^th^ percentile for newborns of the same gestational age (18). Pregnancy hypertension refers to hypertension during pregnancy (19). Placental abruption refers to the condition in which the placenta separates from the uterus during pregnancy (20). Gestational diabetes refers to a kind of hyperglycemia that appears during pregnancy and usually disappears after delivery (21). Abnormal amniotic fluid refers to meconium-stained amniotic fluid and abnormal amniotic fluid level (oligohydramnios or polyhydramnios). Fetal distress refers to the fetus suffering from hypoxia and showing an abnormal fetal heart rate during pregnancy (22). Premature rupture of membranes is defined as a rupture of fetal membranes before labor begins (23). RDS refers to a syndrome of respiratory difficulty caused by a deficiency of surfactants in newborns (24). NEC refers to stage II or III NEC according to the Bell scoring system (25, 26). BPD is defined as a preterm infant with respiratory problems still requiring oxygen support after 28 days of age or past 36 weeks of corrected age (27). PVL refers to a cystic impairment in the periventricular area (28). Sepsis is defined as a severe infection with positive blood culture (29). Severe anemia is defined based on different hemoglobin concentrations at different ages and respiratory status (30).

### Follow-Up

All infants were regularly followed up at least every 3 months after discharge for the assessment of growth and neurodevelopment by experienced pediatric neurologists. The neurodevelopmental outcomes at 18–24 months of corrected age were evaluated according to the Bayley Scales of Infants Development II. Neurological disability was defined as survival with one or more of the following: CP, mental development index (MDI) <70, deafness, and blindness. CP is defined as a group of disorders that impact on movement or posture in childhood (31), and MDI is a comprehensive measure of cognition or language development for evaluating neurodevelopmental outcomes (32). Deafness was defined as total or partial hearing loss, and blindness was defined as a corrected visual acuity worse than 20/200 (33).

### Statistical Analysis

SPSS 23.0 was used for statistical analysis. Categorical data were compared with chi-squared tests or Fisher's exact tests, and continuous data (gestational age and birth weight) were compared with Kruskal–Wallis tests. The non-IVH group was used as controls for *post-hoc* tests when an overall significant difference was seen between the groups. The neurodevelopmental outcomes were compared between the IVH (I-II IVH or III-IV IVH) groups and the non-IVH group using multiple logistic regression. Bilateral α = 0.05 was considered significant.

## Results

**Figure 1:**
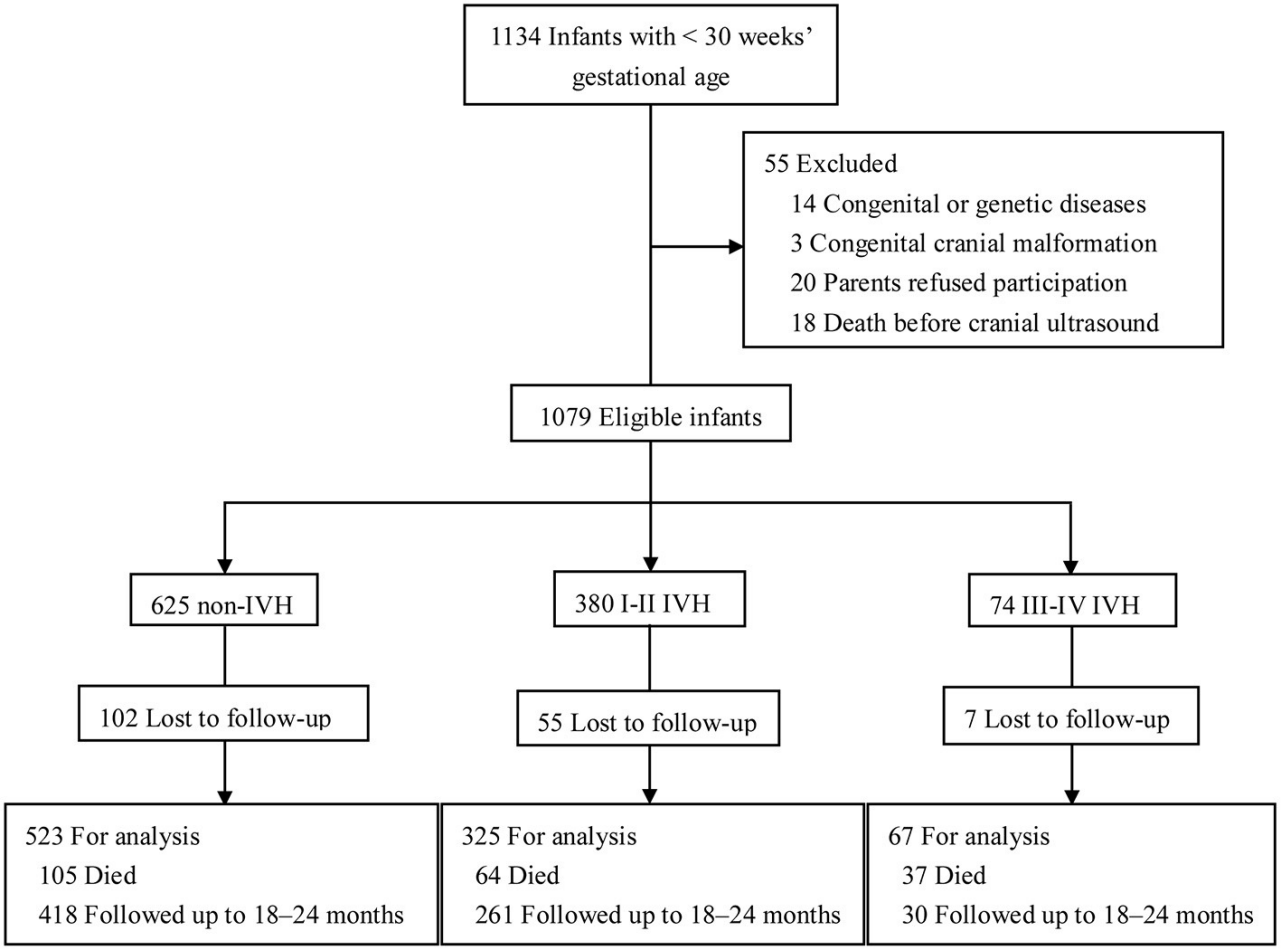
Study flow. Schematic flowchart showing the number of preterm infants in different groups and followed up to 18–24 months of corrected age. Preterm infants were checked with cerebral ultrasound regularly and classified as I-II IVH, III-IV IVH, and non-IVH groups.

There were 1,134 preterm infants admitted into the NICUs during the study period, and 55 of them were excluded (14 with congenital or genetic diseases, 3 with congenital cranial malformation, 20 whose parents refused to participate, and 18 who died before cranial ultrasound examination). Thus, 1,079 preterm infants were eligible (625 without IVH, 380 with I-II IVH, and 74 with III-IV IVH). There were 164 (15.2%) infants lost to follow-up, 206 (19.1%) infants who died, and 915 (84.8%) infants who were followed up to 18–24 months corrected of age (Figure 1). Infants lost to follow-up had significantly lower birth weight and higher rates of maternal age ≥35 years, gestational diabetes, sepsis, BPD, and severe retinopathy of prematurity (ROP) compared to infants followed up to 18–24 months of corrected age (*p* <0.05) (Supplementary Table 1).

In this study cohort, no infants with grade I-II IVH developed hydrocephalus, while the incidence of hydrocephalus in infants with grade III-IV IVH was 15/67 (22.4%). Of nine grade III IVH infants with hydrocephalus (Three of whom died and two of whom developed neurological disabilities), 2 (22.2%) received ventriculoperitoneal shunt. Of 6 grade IV IVH infants with hydrocephalus (Three of whom died and three of whom developed neurological disability), 2 (33.3%) received furosemide therapy to achieve ventricular decompression.

### Perinatal Characteristics

There were no significant differences in birth weight, gender, or the incidence of small for gestational age, 5 min Apgar <4, twin/multiple births, pregnancy hypertension, maternal age ≥35 years, abnormal amniotic fluid, fetal distress, placental abruption, gestational diabetes, or RDS between the groups of preterm infants. Infants with I-II IVH had significantly lower rates of cesarean section birth and higher rates of mechanical ventilation >7 days, sepsis, NEC, BPD, severe ROP, and PVL compared to infants without IVH. Univariate analysis showed that infants with III-IV IVH had significantly lower gestational age, lower rates of cesarean section birth, and higher rates of mechanical ventilation >7 days, sepsis, severe anemia, BPD, ROP, and PVL compared to infants without IVH (Table 1).

**Table 1:** Clinical characteristics of 915 preterm infants.

	**Non-IVH**	**I-II IVH**	**III-IV IVH**	***p*-value**
	**(*n* = 523)**	**(*n* = 325)**	**(*n* = 67)**	
**Neonatal characteristics**				
Gestational age,	28.9 (1.3)	28.9 (1.4)	28.1 (2.0)***	0.000
weeks, median (IQR)				
Birth weight, [g, median (IQR)]	1175 (310)	1180 (295)	1100 (450)	0.351
Male, *n* (%)	303 (57.9)	197 (60.6)	47 (70.1)	0.147
SGA, *n* (%)	22 (4.2)	11 (3.4)	0 (0.0)	0.220
5 min Apgar <4, *n* (%)	35 (6.7)	25 (7.7)	9 (13.4)	0.143
Cesarean section births, *n* (%)	315 (60.2)	149 (45.8)***	28 (41.8)**	0.000
Twin/Multiple births, *n* (%)	148 (28.3)	97 (29.8)	25 (37.3)	0.309
**Maternal characteristics**				
Pregnancy hypertension, *n* (%)	90 (17.2)	48 (14.8)	4 (6.0)	0.051
Maternal age ≥35 years, *n* (%)	106 (20.3)	58 (17.8)	13 (19.4)	0.686
Abnormal amniotic fluid, *n* (%)	76 (14.5)	35 (10.8)	7 (10.4)	0.233
Fetal distress, *n* (%)	92 (17.6)	61 (18.8)	13 (19.4)	0.876
Placental abruption, *n* (%)	44 (8.4)	27 (8.3)	2 (3.0)	0.293
Gestational diabetes, *n* (%)	42 (8.0)	19 (5.8)	2 (3.0)	0.211
Premature rupture of	104 (19.9)	84 (25.8)	21 (31.3)	0.030
membranes, *n* (%)				
**Medical treatment**				
Mechanical ventilation	77 (14.7)	83 (25.5)***	32 (47.8)***	0.000
>7 days, *n* (%)				
EPO treatment, *n* (%)	225 (43.0)	155 (47.7)	20 (29.9)	0.024
**Neonatal complications**				
RDS, *n* (%)	482 (92.2)	306 (94.2)	64 (95.5)	0.388
Sepsis, *n* (%)	131 (25.0)	109 (33.5)**	29 (43.3)**	0.001
Severe anemia, *n* (%)	286 (54.7)	196 (60.3)	47 (70.1)*	0.029
NEC, *n* (%)	21 (4.0)	27 (8.3)**	2 (3.0)	0.018
BPD, *n* (%)	163 (31.2)	151 (46.5)***	38 (56.7)***	0.000
Severe ROP, *n* (%)	8 (1.5)	14 (4.3)*	6 (9.0)**	0.001
PVL, *n* (%)	7 (1.3)	18 (5.5)***	14 (20.9)***	0.000

IQR, interquartile range; SGA, small for gestational age; RDS, respiratory distress syndrome; NEC, necrotizing enterocolitis; ROP, retinopathy of prematurity; BPD, bronchopulmonary dysplasia; PVL, periventricular leukomalacia. ^*^p < 0.05, ^**^p < 0.01, ^***^p < 0.001.

### Neurodevelopmental Outcomes at 18–24 Months of Corrected Age

**Figure 2:**
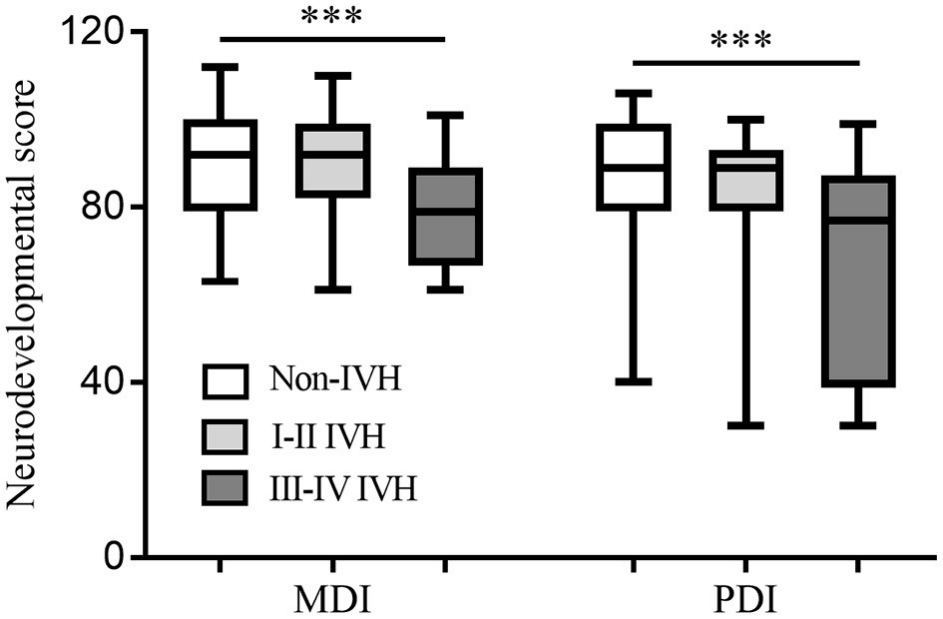
Neurodevelopmental score at 18–24 months of corrected age among the groups. Both MDI and PDI values were significantly lower in the infants with grade III-IV IVH compared to those without IVH. No significant differences were observed between the grade I-II IVH group and the non-IVH. ***p < 0.001, MDI, mental developmental index; PDI, psychomotor development index; IVH, intraventricular hemorrhage.

At 18–24 months of corrected age, the mortality in the non-IVH group, I-II IVH group, and III-IV IVH group was 20.1, 19.7, and 55.2%, respectively (*p* < 0.05), and the incidence of neurodevelopmental disability was 13.9, 16.1, and 43.3%, respectively (*p* < 0.05). The median MDI and PDI values were significantly lower in infants with grade III-IV IVH compared to those without IVH (*p* < 0.001), but no significant differences were observed between the grade I-II IVH group and the non-IVH (*p* > 0.05) (Figure 2).

No significant differences were observed between the I-II IVH group and the non-IVH group in terms of CP, MDI <70, deafness, blindness, disability, death, or disability + death (*p* > 0.05) (Table 2). There were 151 (46.5%) grade I IVH infants and 174 (53.5%) grade II IVH infants in the I-II IVH group, and no significant differences were observed in death (32/151, 21.2% vs. 32/174, 18.4%, *p* = 0.527), disability (18/119, 15.1% vs. 24/142, 16.9%, *p* = 0.697), or disability + death (50/151, 33.1% vs. 56/174, 32.2%, *p* = 0.859) between infants with grade I and grade II IVH. Preterm infants with grade III-IV IVH had significantly higher incidence of CP, MDI <70, disability, death, and disability + death compared to the non-IVH group (*p* <0.05) (Table 2). No significant differences were observed in disability (9/24, 37.5% vs. 4/6, 66.7%, *p* = 0.360), death (26/50, 52.0% vs. 11/17, 64.7%, *p* = 0.363), or disability + death (35/50, 70.0% vs. 15/17, 88.2%, *p* = 0.200) between infants with grade III and grade IV IVH.

**Table 2:** Unadjusted neurodevelopmental outcomes and death at 18–24 months of corrected age.

	**Non-IVH**	**II IVH**	**III-IV IVH**	***p*-value**
	***n*/Total (%)**	***n*/Total (%)**	***n*/Total (%)**	
Cerebral palsy	10/418 (2.4)	11/261 (4.2)	8/30 (26.7)***	0.000
MDI <70	37/418 (8.9)	30/261 (11.5)	9/30 (30.0)**	0.002
Deafness	8/418 (1.9)	7/261 (2.7)	2/30 (6.7)	0.041
Blindness	12/418 (2.9)	5/261 (1.9)	3/30 (10.0)	0.191
Disability	58/418 (13.9)	42/261 (16.1)	13/30 (43.3)***	0.000
Death	105/523 (20.1)	64/325 (19.7)	37/67 (55.2)***	0.000
Disability + death	163/523 (31.2)	106/325 (32.6)	50/67 (73.7)***	0.000

MDI, Mental developmental index; Disability is defined as surviving infants with one or more of the following complications: cerebral palsy, MDI <70, blindness, or deafness. ^**^p < 0.01, ^***^p < 0.001.

Of the 380 grade I-II IVH infants, 261 (68.7%) developed bilateral IVH, and of 74 grade III-IV IVH infants, 58 (78.4%) developed bilateral IVH. Infants with bilateral IVH did not have an increased incidence of death (*p* > 0.05), disability (*p* > 0.05), or death + disability (*p* > 0.05) in either the mild (I-II) or severe (III-IV) IVH groups.

There were 39 infants who developed cystic PVL (7/523 (1.4%) without IVH, 18/325 (5.5%) with grade I-II IVH, and 14 (20.9%) with grade III-IV IVH) according to MRI examinations at 40 weeks of corrected age. Grade I-II IVH infants with cystic PVL had a higher rate of disabilities compared to grade I-II infants without cystic PVL at 18–24 months of corrected age (6/17, 35.3% vs. 36/244, 14.8%, respectively, *p* = 0.038). There were no significant differences in death or death + disability between the two groups (*p* > 0.05).

Ten infants [10/418 (2.4%)] developed CP without IVH (6 infants with mild CP and 4 infants with moderate or severe CP), 11 grade I-II IVH infants (11/216, 5.1%) developed CP (2 infants with mild CP and 9 infants with moderate or severe CP), and 8 grade III-IV IVH infants (8/30, 26.7%) developed CP (all with moderate or severe CP).

**Figure 3:**
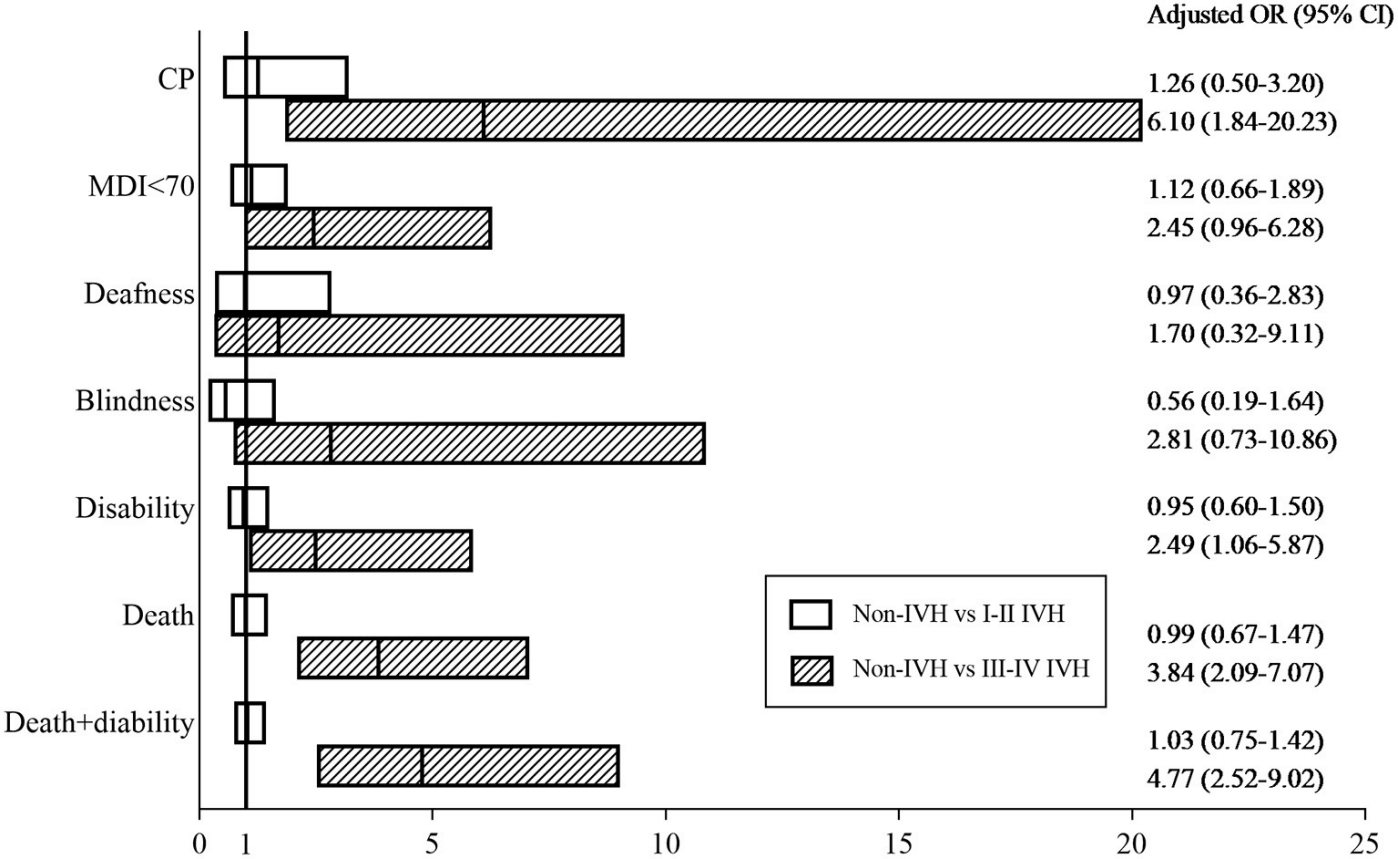
Adjusted odds ratios. Adjusted odds ratios for death and neurological outcomes between the IVH (I-II IVH or III-IV IVH) groups and the non-IVH group at 18–24 months of corrected age. The Hosmer–Lemeshow Test was used to test the goodness-of-fit of the model.

In addition to IVH, confounding factors such as neonatal characteristics, maternal characteristics, medical treatment, neonatal complications, and socioeconomic factors can also impact neurological outcomes and death. After adjusting for confounding factors using multiple logistic analysis, preterm infants with III-IV IVH had higher rates of CP [26.7 vs. 2.4%, OR = 6.10, 95% CI (1.840–20.231), *p* = 0.003], disability [43.3 vs. 13.9%, OR = 2.49, 95% CI (1.059–5.873), *p* = 0.037], death [55.2 vs. 20.1%, OR = 3.84, 95% CI (2.090–7.067), *p* <0.001], and disability + death [73.7 vs. 28.7%, OR = 4.77, 95% CI (2.518–9.021), *p* <0.001] than those without IVH (Figure 3, Supplementary Table 2).

### Subgroup Analysis of Neurodevelopmental Outcomes Between the I-II IVH Group and the Non-IVH Group

No significant differences were observed in terms of disability, death, or disability + death (*p* > 0.05) between preterm infants with I-II IVH and those without IVH either in the 24–28 week's gestation subgroup or the 28–30 week's gestation subgroup. Gestational age had no interaction effect on the impact of I-II IVH on neurological outcomes or mortality in preterm infants born <30 weeks' gestation (Table 3).

**Table 3:** Subgroup analysis the impact of I-II IVH on outcomes at 18–24 months of corrected age.

**Gestation**	**Non-IVH**	**II IVH**	**Adjusted OR**	**Adjusted**
	***n*/Total (%)**	***n*/Total (%)**	**(95% CI)**	***p*-value**
**Disability**				
24–27^+6^ weeks	8/55 (14.5)	12/54 (22.2)	1.467 [0.489–4.396]	0.494
28–29^+6^ weeks	50/363 (13.8)	30/207 (14.5)	0.890 [0.536–1.478]	0.652
**Death**				
24–27^+6^ weeks	38/93 (40.9)	21/75 (28.0)	0.914 [0.434–1.924]	0.812
28–29^+6^ weeks	67/430 (15.6)	43/250 (17.2)	1.097 [0.698–1.725]	0.687
**Disability** **+** **death**				
24–27^+6^ weeks	46/93 (49.5)	33/75 (44.0)	0.803 [0.429–1.501]	0.491
28–29^+6^ weeks	117/430 (27.2)	73/250 (29.2)	1.225 [0.785–1.913]	0.372

*Disability is defined as surviving infants with one or more of the following complications: cerebral palsy, MDI <70, blindness, or deafness*.

## Discussion

This very preterm cohort showed that mortality and neurodevelopmental outcomes are different according to the severity of IVH in very preterm infants. Preterm infants with I-II IVH and <28 weeks' gestation and those with 28–30 weeks' gestation had similar neurological outcomes and mortality compared to those without IVH. However, III-IV IVH was associated with CP, disability, mortality, and disability + death at 18–24 months of corrected age.

Grade I-II IVH occurs in the germinal matrix or ventricles without ventricular dilatation. The impact of I-II IVH on long-term neurological outcomes in preterm infants has always been a concern because of the conflicting results reported on this issue. In the current study, infants with I-II IVH did not show increased adverse neurological outcomes compared with the non-IVH group after adjusting for confounding factors, and this was consistent with some previous clinical studies (Supplementary Table 3) (10, 16, 34). Even though infants with grade I-II IVH had higher rates of neonatal morbidities such as sepsis, NEC, BDP, severe ROP, and PVL compared with infants with non-IVH (Table 1), BPD and PVL were independent risk factors for disability (Supplementary Table 2). However, it is not grade I-II IVH itself that results in higher rates of neonatal morbidities, and instead multiple perinatal risk factors lead to a simultaneous increase in the incidence of grade I-II IVH, BPD, and PVL in very preterm infants. Reubsaet et al. (16) performed a study with a cohort of 342 preterm infants with a gestational age of 24–32 weeks, and they found that preterm infants with I-II IVH had similar neurodevelopmental outcomes compared with controls at 2 years' corrected age. With a lager sample size, our study came to a similar conclusion at 18–24 months' corrected age. A large sample size, multi-center cohort including 1,472 preterm infants with an extremely low gestational age (<27 weeks' gestation) also found that low-degree IVH had no effect on neurological outcomes (10). Ann Wy et al. (34) enrolled 985 premature infants (gestational age <37 weeks) and found that I-II IVH was not associated with adverse outcomes in intelligence, academic achievement, or problematic behavior at 3, 8, and 18 years of age. Thus, grade I-II IVH might not impair neurological development in preterm infants.

In contrast to those studies, however, several other studies have shown adverse neurodevelopmental outcomes in preterm infants with I-II IVH (Supplementary Table 3) (15, 35, 36). It has been reported that I-II IVH is followed by subtle impairment in subcortical white matter development, which has relevant impacts on the development of processing skills and fine motor coordination, especially in preterm infants with low gestational age (12, 13). Patra et al. (35) found that I-II IVH was associated with adverse neurological outcomes in extremely preterm infants at 20 months of corrected age even without any white matter abnormalities. The adverse neurological outcomes in infants assessed at the first years of life are more likely to be associated with cognitive and neurological impairment at school age (37). With a routine long-term follow-up, Klebermass-Scherhof et al. (36) found that I-II IVH was associated with significant adverse neurological outcomes at 1, 2, 3, and 5.5 years of age. Hollebrandse et al. (9) showed that I-II IVH was associated with a significantly higher rate of CP in school-aged children born extremely preterm, but not with any difficulties in academic skills or motor dysfunction. Some studies support the finding that reduced cortical gray matter following I-II IVH may result in neurological disabilities in preterm infants (36, 38). The reduction in cortical gray matter might be due to glial precursor cell loss during migration, and the absence of astrocytes and oligodendrocytes might then disrupt cerebral cortical development and myelination (36).

Severe IVH with ventricular dilatation or parenchymal involvement is well-known to be related to poor outcomes in preterm infants (39). In our study, III-IV IVH increased the incidence of neurological disability, which was consistent with other studies (15, 36, 40, 41). Ventricular dilatation, periventricular edema, and increased intracranial pressure following the pathological process of III-IV IVH all contribute to the development of neurological disability (15, 42). Oxygen deprivation due to persistent inhibition of cerebral blood flow, hemoglobin-mediated oxidative injury, and cell death following III-IV IVH also contribute to the development of neurological disability (43–45). In this study, grade III IVH and grade IV IVH were combined into a single entity because both groups of IVH are associated with poor outcomes. There was a tendency for a higher rate of disability or death in grade IV IVH compared to grade III, but the difference was not statistically significant. This might be related to lower numbers of infants with grade IV IVH. Due to different pathogenic mechanisms, grade IV IVH presumably represents a different and more severe form of injury compared to grade III.

Mortality in preterm infants with IVH is associated with the severity of bleeding, and III-IV IVH is one of the main causes of death in preterm infants (46). In our study, preterm infants with III-IV IVH had a significant increase in mortality compared to those without IVH. Preterm infants with III-IV IVH had a higher rate of post-hemorrhagic hydrocephalus, and some of them required surgical shunts to decrease intracranial pressure, all of which contribute to the development of neurological disability and death (47). However, there are currently no effective therapies for neonatal IVH. Fortunately, in the past few decades antenatal steroids (48), magnesium sulfate (49), mesenchymal stem cells (50), and erythropoietin (30) have shown promise as therapeutic agents for neuroprotection against death and neurological disability in preterm infants with severe IVH.

There were some limitations in this study. First, preterm infants who were lost to follow-up had higher rates of neonatal complications such as sepsis, BPD, and ROP than those who completed follow-up (Supplementary Table 1), which might lead to more preterm infants with poor neurological outcomes being lost to follow-up. Second, the follow-up at 18–24 months of corrected age limited the power of our study. Subsequent follow-ups to school age or even longer for comprehensively evaluating the impact of IVH on neurological outcomes in preterm infants are needed.

## Conclusions

Our study indicated that preterm infants of <30 weeks' gestational age with I-II IVH had similar neurological outcomes and mortality compared to those without IVH, while III-IV IVH was associated with CP, disability, death, and disability + death at 18–24 months of corrected age. Effective strategies are required to protect against III-IV IVH and reduce adverse neurological outcomes. Further follow-up to school age is also needed in preterm infants with IVH.

## Data Availability Statement

The raw data supporting the conclusions of this article will be made available by the authors, without undue reservation.

## Ethics Statement

The studies involving human participants were reviewed and approved by the Ethics Committee of the Third Affiliated Hospital of Zhengzhou University. Written informed consent to participate in this study was provided by the participants' legal guardian/next of kin.

## Author Contributions

YW and CZ designed the study. YW, JS, XZ, WK, WL, YY, SZ, and FX were involved in data collection. YW, JS, XW, and CZ analyzed the data and wrote the paper. All authors have read and approved the final manuscript.

## Funding

This study was supported by National Key Research and Development Program of China (2018YFC1004604), the National Nature Science Foundation of China (U21A20347), Swedish Research Council (2018-02267), ALF (ALFGBG-965197), The Royal Society for Science and Knowledge in Gothenburg (2020-476, 2021-496), and Stiftelsen Edit Jacobsons Donationsfond (2021-102).

## Conflict of Interest

The authors declare that the research was conducted in the absence of any commercial or financial relationships that could be construed as a potential conflict of interest.

## Publisher's Note

All claims expressed in this article are solely those of the authors and do not necessarily represent those of their affiliated organizations, or those of the publisher, the editors and the reviewers. Any product that may be evaluated in this article, or claim that may be made by its manufacturer, is not guaranteed or endorsed by the publisher.
