# Supplementary material for: The Impact of Different Degrees of Intraventricular Hemorrhage on Mortality and Neurological Outcomes in Very Preterm Infants: A Prospective Cohort Study
Source: Front Neurol. 2022 Mar 21;13:853417. doi: 10.3389/fneur.2022.853417 (PMC8978798; doi:10.3389/fneur.2022.853417)
Supplement: Supplementary file 1 [file Table_1.DOCX]

**Suppl. table1.** Clinical characteristics between infants followed up and lost to follow-up

|  | Followed up  (n = 915) | Lost to follow-up  (n = 164) |
| --- | --- | --- |
| **Neonatal Characteristics** |  |  |
| Gestational age, (weeks, median (IQR)) | 28.6 (1.4) | 28.4 (1.7) |
| Birth weight, (g, median (IQR)) | 1170 (300) | 1100 (350)* |
| Male, n (%) | 547 (59.8) | 92 (56.1) |
| SGA, n (%) | 33 (3.6) | 5 (3.0) |
| 5 min Apgar <4, n (%) | 69 (7.5) | 11 (6.7) |
| Caesarean section births, n (%) | 492 (53.8) | 88 (53.7) |
| Two/Multiple births, n (%) | 270 (29.5) | 51 (31.1) |
| **Maternal Characteristics** |  |  |
| Pregnancy hypertension, n (%) | 142 (15.5) | 28 (17.1) |
| Maternal age, ≥35 years, n (%) | 177 (19.3) | 48 (29.3)** |
| Abnormal amniotic fluid, n (%) | 118 (12.9) | 22 (13.4) |
| Fetal distress, n (%) | 166 (18.1) | 33 (20.1) |
| Placental abruption, n (%) | 73 (8.0) | 9 (5.5) |
| Gestational diabetes, n (%) | 63 (6.9) | 24 (14.6)** |
| Premature rupture of membranes, n (%) | 209 (22.8) | 48 (29.3) |
| **Medical Treatment** |  |  |
| Mechanical ventilation >7 days, n (%) | 192 (21.0) | 44 (26.8) |
| Erythropoietin treatment, n (%) | 400 (43.7) | 79 (48.2) |
| **Neonatal Complications** |  |  |
| RDS, n (%) | 852 (93.1) | 156 (95.1) |
| Sepsis, n (%) | 269 (29.4) | 65 (39.6)** |
| Severe anemia, n (%) | 529 (57.8) | 101 (61.6) |
| NEC, n (%) | 50 (5.5) | 9 (5.5) |
| BPD, n (%) | 345 (37.7) | 91 (55.5)*** |
| Severe ROP, n (%) | 28 (3.1) | 24 (14.6)*** |
| PVL, n (%) | 39 (4.3) | 11 (6.7) |
| IVH |  |  |
| I-II IVH, n (%) | 325 (35.5) | 55 (33.5) |
| III-IV IVH, n (%) | 67 (7.3) | 7 (4.3) |

Note: SGA: small for gestational age; RDS: respiratory distress syndrome; NEC: necrotizing enterocolitis; BPD: bronchopulmonary dysplasia; ROP: retinopathy of prematurity; PVL: periventricular leukomalacia. *P < 0.05, **P < 0.01, ***P < 0.001.
