# Supplementary material for: The Impact of Different Degrees of Intraventricular Hemorrhage on Mortality and Neurological Outcomes in Very Preterm Infants: A Prospective Cohort Study
Source: Front Neurol. 2022 Mar 21;13:853417. doi: 10.3389/fneur.2022.853417 (PMC8978798; doi:10.3389/fneur.2022.853417)
Supplement: Supplementary file 2 [file Table_2.docx]

**Supplementary table 2. Logistic models for the impact of different grade of IVH on mortality and neurological outcomes.**

| Outcomes No.% | CF | B | SE | Wald | df | P | Exp(B)(95%CI) | Hosmer-Lemeshow Test | |
| --- | --- | --- | --- | --- | --- | --- | --- | --- | --- |
| CP, 29/709 (4.1%) | 5 min Apgar <4 | 2.113 | 0.575 | 13.49 | 1 | 0.000 | 8.27(2.68-25.54) | P=0.65 |  |
|  | PRM | 1.185 | 0.436 | 7.39 | 1 | 0.007 | 3.27(1.39-7.69) |  |  |
|  | EPO treatment | -0.920 | 0.440 | 4.37 | 1 | 0.037 | 0.40(0.17-0.94) |  |  |
|  | BPD | 0.972 | 0.438 | 4.93 | 1 | 0.026 | 2.64(1.12-9.23) |  |  |
|  | PVL | 1.796 | 0.556 | 10.44 | 1 | 0.001 | 6.02(2.03-17.90) |  |  |
| Disability,  113/709 (15.9%) | BPD | 0.632 | 0.217 | 8.50 | 1 | 0.004 | 1.88(1.23-2.88) | P=0.75 |  |
|  | PVL | 1.170 | 0.404 | 8.39 | 1 | 0.004 | 3.22(1.46-7.11) |  |  |
| Death,  206/915 (22.5%) | Gestational age | -0.317 | 0.102 | 9.63 | 1 | 0.002 | 0.73(0.59-0.89) | P=0.97 |  |
|  | Birth weight | -0.002 | 0.000 | 11.25 | 1 | 0.001 | 0.99(0.99-0.99) |  |  |
|  | 5 min Apgar <4 | 1.076 | 0.297 | 13.11 | 1 | 0.000 | 2.93(1.64-5.25) |  |  |
|  | PRM | 0.421 | 0.206 | 4.18 | 1 | 0.041 | 1.52(1.02-2.28) |  |  |
|  | EPO treatment | -1.189 | 0.200 | 35.47 | 1 | 0.000 | 0.31(0.21-0.45) |  |  |
| Disability+death,  319/915 (34.9%) | Gestational age | -0.290 | 0.086 | 11.29 | 1 | 0.001 | 0.75(0.63-0.89) | P=0.94 |  |
|  | Birth weight | -0.001 | 0.000 | 6.20 | 1 | 0.013 | 0.99(0.99-1.00) |  |  |
|  | 5 min Apgar <4 | 0.845 | 0.281 | 9.03 | 1 | 0.003 | 2.33(1.34-4.04) |  |  |
|  | EPO treatment | -0.667 | 0.155 | 18.47 | 1 | 0.000 | 0.51(0.38-0.69) |  |  |
|  | Severe anemia | 0.356 | 0.157 | 5.10 | 1 | 0.024 | 1.43(1.05-1.94) |  |  |

Note: Hosmer and Lemeshow Test was used to test the goodness-of-fit of the model. p > 0.05 means a model fit the data well. PRM: Premature rupture of membranes; EPO: Erythropoietin; CF: Confounding factors; CP: cerebral palsy; BPD: bronchopulmonary dysplasia; PVL: periventricular leukomalacia. CI: confidence interval; SE: standard error; Wald: Wald test. df: degree of freedom。
