# Supplementary material for: The Impact of Different Degrees of Intraventricular Hemorrhage on Mortality and Neurological Outcomes in Very Preterm Infants: A Prospective Cohort Study
Source: Front Neurol. 2022 Mar 21;13:853417. doi: 10.3389/fneur.2022.853417 (PMC8978798; doi:10.3389/fneur.2022.853417)
Supplement: Supplementary file 3 [file Table_3.docx]

**Supplementary table 3. Summary of the prognosis of I-II IVH in previous studies.**

| Author, Year | | Exclusion Criteria | Gestational age | | Definition of IVH | Neurodevelopmental outcomes | Age of assessment |
| --- | --- | --- | --- | --- | --- | --- | --- |
| **Nomal outcomes** | | | | | | | |
| Pauline, 2017 | Congenital abnormalities, PVL  III-IV IVH, cerebellar hemorrhage,  focal infarction | | 24-32 weeks | Papile’s classification system,  examination by CUS and MRI | | No significant differences in CP, neurodevelopmental delay,  visual and hearing impairment | 18-30 months’ corrected age |
| Payne, 2013 | Major congenital anomalies,  hydrocephalus requiring shunt,  meningitis and porencephalic cyst | | < 27 weeks | Papile’s classification system,  examination by CUS | | No significant differences in CP, neurodevelopmental delay,  visual and hearing impairment | 18-22 months’ corrected age |
| Ann, 2015 |  | | < 37 weeks | Papile’s classification system,  examination by CUS | | No significant differences in cognitive function, behavior and academic achievement | 3,8 and 18 years of age |
| **Adverse outcomes** | | | | | | | |
| Bolisetty, 2014 | Congenital abnormality | | 23-28 weeks | Papile’s classification system,  examination by CUS | | Significantly worse outcomes in CP, neurosensory impairment,  MDI or GQ≤2 SD and deafness | 2-3 years’ corrected age |
| Patra, 2006 | PVL, III-IV IVH, meningitis, VP shunt, lenticulostriate vasculopathy, ventricular dilation and porencephaly | | 26.3±1.8 weeks | Papile’s classification system,  examination by CUS | | Significantly worse outcomes in MDI<70, neurologic abnormality and neurological impairment. | 20 months’ corrected age |
| Klebermass-Scherhof, 2012 | periventricular leucomalacia and cerebellar lesions | | <32 weeks | Papile’s classification system,  examination by CUS | | Significantly worse outcomes in PDI, CP and visual impairment | 1,2,3 and5.5 years of age |

Note: CUS: cranial ultrasound; IVH: intraventricular hemorrhage; MRI: magnetic resonance imaging; PDI: psychomotor developmental index; MDI: mental developmental index; PVL: periventricular leukomalacia; VP: ventriculoperitoneal
